# Characterization of a loss-of-function NSF attachment protein beta mutation in monozygotic triplets affected with epilepsy and autism using cortical neurons from proband-derived and CRISPR-corrected induced pluripotent stem cell lines

**DOI:** 10.3389/fnins.2023.1302470

**Published:** 2024-01-08

**Authors:** Gowher Ali, Kyung Chul Shin, Wesal Habbab, Ghaneya Alkhadairi, Alice AbdelAleem, Fouad A. AlShaban, Yongsoo Park, Lawrence W. Stanton

**Affiliations:** ^1^Neurological Disorders Research Center, Qatar Biomedical Research Institute (QBRI), Hamad Bin Khalifa University (HBKU), Qatar Foundation, Doha, Qatar; ^2^Medical Molecular Genetics Department, Human Genetics and Genome Research Institute, National Research Centre, Cairo, Egypt; ^3^College of Health and Life Sciences (CHLS), Hamad Bin Khalifa University (HBKU), Qatar Foundation, Doha, Qatar

**Keywords:** cortical neuron, NAPB, iPSC, CRISPR/Cas9, exon skipping

## Abstract

We investigated whether a homozygous recessive genetic variant of NSF attachment protein beta (*NAPB*) gene inherited by monozygotic triplets contributed to their phenotype of early-onset epilepsy and autism. Induced pluripotent stem cell (iPSC) lines were generated from all three probands and both parents. The *NAPB* genetic variation was corrected in iPSC lines from two probands by CRISPR/Cas9 gene editing. Cortical neurons were produced by directed, *in vitro* differentiation from all iPSC lines. These cell line-derived neurons enabled us to determine that the genetic variation in the probands causes exon skipping and complete absence of NAPB protein. Electrophysiological and transcriptomic comparisons of cortical neurons derived from parents and probands cell lines indicate that loss of NAPB function contributes to alterations in neuronal functions and likely contributed to the impaired neurodevelopment of the triplets.

## Introduction

We have performed genome sequencing on more than 100 families in Qatar comprised of children presenting with autism spectrum disorder (ASD) and other neurodevelopmental comorbidities. By sequencing the genomes of both parents and the affected proband (s), this study has discovered dozens of *de novo* and inherited genetic variants that are candidate risk genes for ASD and other rare neurodevelopmental disorders (unpublished data). One recruited family of Palestinian ancestry was comprised of first-cousin consanguineous parents, monozygotic triplet probands, and five non-affected offspring. Comprehensive clinical assessment of the probands was reported elsewhere (AbdelAleem et al., 2023). Briefly, all probands had mild facial dysmorphisms, learning disabilities, and motor developmental delays. Epilepsy was prominent in all three probands from a young age and persists into adulthood (now 23 years of age) with varying degrees of severity and frequency. Interestingly, autism spectrum disorder was severe in one proband, mild in another and absent in the third.

Whole exome sequencing revealed two homozygous autosomal genetic variants of interest in all three probands, inherited from their consanguineous parents who were heterozygous for both genes (our unpublished data) and reported independently elsewhere (AbdelAleem et al., 2023). One variant is a missense mutation in exon 46 of *VPS13B*, c.8516G>A 9 (p.Arg2839Gln) on chromosome 8. The other is a splice site loss variant, c.354+2T>G, in intron 4 of *NAPB* on chromosome 20. It is predicted that this splice site loss would result in exon skipping of 47 bp exon 4, thereby creating a frame shift that produces a truncated or completely lost version of the 298 amino acid NAPB protein.

*VPS13B* encodes vacuolar protein sorting 13b, a large protein that plays a role in membrane vesicle trafficking in the Golgi (Seifert et al., 2011). The missense variant found in the probands is potentially deleterious given that it is homozygous with a CADD score of 33. Loss-of-function (LoF) mutations in *VPS13B* have been previously linked to Cohen syndrome (Kolehmainen et al., 2003; Mochida et al., 2004; Momtazmanesh et al., 2020; Li et al., 2021), a rare disorder associated with developmental delay, facial dysmorphisms, and learning disabilities, a spectrum of features seen in the triplet probands (Kivitie-Kallio and Norio, 2001). However, the early onset of epilepsy observed in the probands was not a reported feature of Cohen syndrome, and other features of Cohen syndrome were absent, suggesting that additional genetic variants in the triplets are contributing to the unique clinical phenotype.

Here we report our investigation of the *NAPB* genetic LoF variant. NAPB, N-ethylmaleimide-sensitive factor attachment protein beta, is expressed primarily in the brain and plays a role in synaptic vesicle recycling. NAPB serves as a co-factor of NSF ATPase during SNARE complex disassembly, inducing increased levels of the free SNARE components for subsequent fusion reactions (Burgalossi et al., 2010; Rizzoli, 2014). There is accumulating evidence that SNARE proteins and SNARE-regulatory proteins play an important role in neuronal regulation and brain development (Wojcik and Brose, 2007). Protein truncating variants of NAPB have been previously identified in cases of early onset epileptic encephalopathy (EOEE) (Conroy et al., 2016; Zhao et al., 2021; Mignon-Ravix et al., 2023). The novel, homozygous inherited variant we discovered in the Palestinian triplets results in the loss of splice donor site and presumably null expression of NAPB protein.

To investigate the molecular and biological consequence of NAPB LoF in human neurons, we generated induced pluripotent stem cell (iPSC) lines from all three probands and their parents (Ali et al., 2022). Isogenic iPSC lines were also generated by CRISPR gene editing to correct the *NAPB* mutation in proband-derived iPSC. We present here the derivation and characterization of cortical human neurons from these patient-specific iPSC lines. This cell-based model afforded the opportunity for us to perform electrophysiological and transcriptomic comparisons between wild-type (parents) and NAPB LoF (probands) human neurons.

## Materials and methods

### Stem cell differentiation into cortical neurons

In the present study, we used induced pluripotent stem cells (iPSCs) derived from monozygotic triplets having homozygous splice donor mutation in *NAPB* (c.354+2T>G) and missense variant in *VPS13B* c.8516G>A 9 (p.Arg2839Gln) with varying degree of neurodevelopmental disorder (NDD) and their heterozygous parents (Ali et al., 2022; AbdelAleem et al., 2023). Parents derived control iPSCs (CtrlF and CtrlM) and probands derived iPSCs (NDD_01, NDD_04 and NDD_05) were maintained under a feeder free condition in mTeSR1 medium (Stemcell Technologies, Vancouver) on Matrigel (1:80, BD Biosciences) coated plate cultured in a 37°C incubator with humidified atmosphere and 5% CO_2_. Colonies were passaged as small clumps using Gentle cell dissociation reagent (Stemcell Technologies Technologies).

Control and mutant iPSCs were differentiated into cortical neurons following the published protocols (Shi et al., 2012), with some modifications. Briefly, iPSCs colonies were washed with PBS to remove dead cells and dissociated into single cells using TrypLE (Thermo Fisher Scientific). The single cells were plated onto matrigel coated plates in mTeSR1 medium containing 10 μM Y-276321 (ROCK inhibitor). Next day, the cells were 90%–100% confluent and differentiation was initiated by changing medium to Neurobasal medium (DMEM/F12, Neurobasal, 1X B-27 minus vitamin A, 1X N2 supplement, 1X L-Glutamine, 1X Non-essential amino acids (NEAA), 50 μM β-mercapto-ethanol, 0.2X Penicillin/streptomycin) supplemented with dual SMAD inhibitors10 μM SB431542 and 2 μM Dorsomorphin for 12 days. During neural induction, the cells were split at day 8 and 12 using TrypLE and plated onto Matrigel-coated plates in neurobasal media containing 5 μM Rock inhibitor. For neural proliferation (days 14–18), the neurobasal media was supplemented with 20 ng/mL bFGF. At day 20, neural progenitor cells (NPCs) were cryopreserved or plated for maturation onto Matrigel-coated plates in neurobasal media supplemented with 10 ng/mL BDNF, 10 ng/mL GDNF, 2 μg/mL insulin, 20 μM dibutyryl-cyclic AMP (db-cAMP, Sigma), and 200 μM Ascorbic acid (AA, Sigma). On day 28, the cells were plated for experiment at a density of 50,000 cells/cm2 onto 100 μg/mL poly-L-ornithine (PO, Sigma) and 20 μg/mL laminin-coated plates (Invitrogen) and media was changed every 2–3 days. The cells were allowed to mature for 8 weeks.

### Immunostaining

Cells were washed with 1X PBS and fixed with 4% paraformaldehyde for 20 min at room temperature. The fixed cells were washed three times with PBS, treated with 0.2% Triton X-100 (Sigma-Aldrich) in PBS for 30 min to permeabilize and blocked in PBST (PBS with 0.2% tween-20) containing 3% bovine serum albumin (BSA) for 2–3 h. The cells were incubated with primary antibodies overnight at 4°C. Primary antibodies consisted of PAX6 (Mouse, 1:100, Abcam: ab78545), SOX2 (Rabbit, 1:200, Invitrogen: MA1-014), OTX2 (Goat, 1:300, R&D: AF1979), FOXG1 (Rabbit, 1:200, Abcam: ab18259), Nestin (Mouse, 1:100, Invitrogen: MA1110), MAP2 (chicken, 1:500, Abcam: ab5392), MAP2 (Mouse, 1:500, Invitrogen: 13-1500), CTIP2 (Rabbit, 1:200, Cell Signaling), TBR1 (Rabbit,1:200, Invitrogen: PA5-34582), BRN2 (Rabbit, 1:200, Cell Signaling: 12137), GFAP (Chicken, 1:400, Abcam: ab4674), SATB2 (Rabbit, 1:200, Invitrogen: PA5-83092), anti-GABA (Rabbit, 1:200, Sigma: A2052), vGLUT1 (Guinea pig, 1:200, Synaptic system: 135304), PSD-95 (Mouse, 1:500, Invitrogen: 51-6900), anti-synaptophysin (Rabbit, 1:200, Abcam: ab32127). Next day, the cells were washed three times with PBST at 10 min intervals and incubated with the secondary antibodies diluted 1:1000 in PBST containing 3% BSA for 1 h at room temperature. Secondary antibodies were conjugated with Alexa Flour 488, Alexa Flour 555, and Alexa Flour 647 dyes (Thermo Fisher Scientific). Nuclei were stained with DAPI (Thermo Fisher Scientific) for 5 min. Cells were washed three times with PBS and imaged using the inverted fluorescence microscope (Olympus IX 53).

### Western blot

The cells were washed with PBS and lysed in Laemmle sample buffer (200 mM Tris-HCl, pH 6.8, 8% sodium dodecyl sulfate, 0.4% Bromophenol blue, 20% glycerol, 5% 2-mercaptoethanl). The lysates were sonicated and heated at 95°C for 10 min. The protein samples were loaded and separated using sodium dodecyl sulfate polyacrylamide gel electrophoresis (SDS-PAGE) and transferred to nitrocellulose membrane (Bio-Rad, Cat#1620112). Blots were then blocked with 5% skim milk in PBST (PBS containing 0.2% Tween-20) for at least 2–3 h at room temperature and Immunoblotting was performed overnight at 4°C with the following antibodies: NAPB antibody (Abcam ab228771, 1:1000), GAPDH (Abcam: 1:2000) The blots were washed the next day and incubated with Goat anti-Rabbit-HRP secondary antibody (Cat# 31460, Thermo Fisher Scientific, 1:3000). The Protein bands were subsequently scanned using the ChemiDoc imaging system (BioRad).

### CRISPR/Cas9 editing

For editing, the guide RNA (gRNA) sequence targeting the region surrounding the *NAPB* mutation was selected using CRISPR-Cas9 gRNA design tool (Integrated DNA technologies). Single guide RNA (sgRNA) was synthesized using EnGen sgRNA Synthesis Kit (NEB, E3322) according to the manufacturer’s instructions. Nucleofection was carried out using the Amaxa nucleofection system (P3 primary cell 4D-nucleofector kit, Cat#V4XP-3032) according to the manufacturer’s instructions. Briefly, RNP complex were generated by mixing 1 μg of sgRNA with 2 μM of EnGen SpyCas9 NLS (NEB, M0646) at room temperature for 15–20 min. Approximately 2.5–3 × 10^5^ iPSCs were electroporated using CB150 nucleofection program and plated onto Matrigel-coated plates. Approximately 2 μg of knock-in oligo (Supplementary Table S1) was added to the nucleofection mix just before nucleofection. The cleavage efficiency of sgRNA was evaluated using T7E1 cleavage assay. After 48 h the cells were diluted and plated as single cells on Matrigel-coated plates for 10–15 days to make colonies. Genomic DNA (gDNA) was extracted using quick extract genomic DNA extraction buffer (epicenter). The region of *NAPB* targeted by sgRNA was amplified with specific primers (Supplementary Table S1) using PCR-Master mix (Thermo Fisher Scientific) and knock-in was confirmed by sanger sequencing of the PCR product.

### Electrophysiology

The whole-cell patch-clamp technique was used for recording evoked action potentials using an EPC-10 USB amplifier (HEKA Elektronik, Lambrecht/Pfalz, Germany) with the software (HEKA Patchmaster), filtering at 5 kHz and sampling at 10 kHz. Evoked action potentials were recorded by a series of current steps from 20 to 60 pA for 500 ms in a current-clamp mode. Whole-cell currents were measured by a series of 20 mV voltage steps from 120 to 60 mV for 1 s in a voltage-clamp mode. Neurons were cultured in the coverslip and placed in the chamber to be perfused with the normal Tyrode’s (NT) bath solution (mM): 143 NaCl, 5.4 KCl, 0.33 NaHPO_4_, 0.5 MgCl_2_, 5 HEPES, 2 CaCl_2_, and 11 glucose; pH 7.4 adjusted with NaOH. The internal pipette solution contained (mM): 130K-gluconate, 3 KCl, 2 MgCl_2_, 10 HEPES, 5 Na_2_ATP, 0.5 Na_2_GTP, 0.2 EGTA; pH 7.3 adjusted with KOH. Patch pipettes were pulled from borosilicate capillary tubes (A-M systems, WA, United States) using a puller PC-10 (Narishige, Tokyo, Japan). Final resistance of the electrode pipette tips was 3–5 MΩ.

### Calcium imaging

The neurons on coverslips were loaded with 3 μM Fura-2 AM (Thermo Fisher Scientific) for 30 min at room temperature in normal Tyrode’s buffer solution above. Calcium imaging experiments were conducted using a monochromator-based spectrofluorometric system (Photon Technology International, Lawrenceville, NJ) with Evolve 512 camera (Teledyne Photometrics, AZ, USA). Dual excitation and emission were at 340/380 and 510 nm, respectively. Data acquisition was accomplished using EasyRatioPro software. 50 mM KCl was applied to depolarize the membrane potential to evoke calcium influx through voltage-gated calcium channels (VGCCs). Regions of interest (ROI) were assigned by highlighting the perimeter of the cell using the software. Mean fluorescence intensity was recorded within the ROIs. Images of representative cells were analyzed after background subtraction for higher accuracy and improved visualization using ImageJ software (National Institutes of Health, Bethesda, MD).

### RNA extraction, real-time PCR and library preparation

For RNA extraction, the cells were lysed in TRIzol (Thermo Fisher Scientific) and total RNA was purified using Direct-zol RNA MiniPrep Extraction Kit (Zymo Research) according the manufacturer’s instructions. Complementary DNA (cDNAs) were synthesized from 0.5 μg of RNA using RevertAid First Strand cDNA Synthesis kit (Thermo Fisher Scientific). Quantitative PCR (qPCR) was performed using Syber Green PCR Master Mix (Applied biosystems) and amplification was detected using Quant Studio 7 system (Applied Biosystems). Gene expression was normalized to GAPDH. The primer details were listed in Supplementary Table S1.

For library preparation, total RNA with an RNA integrity number (RIN) above 8 was used as input using TruSeq Stranded mRNA kit (Cat #: 20020594) from Illumina following the manufacturer’s protocol. Briefly, 0.5 μg of total RNA was used to capture mRNA molecules using poly-T oligo attached magnetic beads and then mRNA was fragmented. cDNA was generated from the cleaved RNA fragments using random priming during first and second strand synthesis. Barcoded DNA adapters were ligated to both ends of DNA, and then amplified. The quality of library generated was checked on an Agilent 2100 Bioanalyzer system and quantified using a Qubit system. Libraries that pass quality control was pooled, clustered on a cBot platform, and sequenced on an Illumina HiSeq 4000 at a minimum of 20 million paired end reads (2 × 75 bp) per sample.

### RNA-seq data analysis

For RNA-seq analysis, the paired-end reads were trimmed using Cutadapt with default parameters to discard low quality reads and trim adaptor sequences. The high-quality reads from each sample were aligned to the Human reference genome (GRCh38/hg38) using STAR version 2.7.10 (Dobin et al., 2013). Transcript counting was carried out using Subread:featureCounts version 2.0.1 (Liao et al., 2014). All gene level transcript counts were imported in R and differential expression analysis was performed with DESeq2 (Love et al., 2014). Genes with adjusted *p*-values <0.05 and fold changes >2 were considered as differentially expressed. The volcano plot and heatmap were created using the ggplot2 and Pheatmap R-libraries, respectively. Gene Ontology (GO) enrichment analysis was performed using ShinyGO package (Ge et al., 2020).

### Statistical analysis

Data analysis was performed using GraphPad Prism 9 (GraphPad Software, San Diego, CA, United States). Data are means ± standard error of the mean (S.E.M.). Data were analyzed using one-way analysis of variance (ANOVA) with Tukey test. Probabilities of *p* < 0.05 was considered significant.

## Results

### Generation of parent and proband-specific iPSC lines

Whole exome sequencing of the parents and their monozygotic triplets, part of a Qatari cohort recruited for an autism genetics study, identified two genetic variants of potential interest: a missense mutation in *VPS13B*, c.8516G>A 9 (p.Arg2839Gln); and a splice site loss variant in *NAPB*, c.354+2T>G. A new blood draw was taken from the three probands, their parents and five unaffected siblings. Targeted sequencing of variant regions of *VPS13B* and *NAPB* confirmed that the parents were heterozygous for both genes and the probands homozygous for the variant alleles. The unaffected siblings were heterozygous or homozygous wild-type for both genes (Supplementary Figures S1A,B), thus variants of both *VPS13B* and *NAPB* present as plausible candidates for consideration as causative for the neurodevelopmental phenotype observed in the probands.

Peripheral blood mononuclear cells extracted from the probands and both parents were transduced with non-integrating Sendai virus expressing OCT4, SOX2, c-MYC and KLF4 transcription factors to generate induced pluripotent stem cell (iPSC) lines for each subject (Ali et al., 2022). Selected iPSC lines had typical human embryonic stem cell (hESC)-like morphology, expressed defining markers of pluripotency, and differentiated into all three germ layers *in vitro* (Ali et al., 2022). Targeted sequencing of genomic DNA confirmed the expected genotypes of *VPS13B* and *NAPB* in all iPSC lines (Supplementary Figures S1A,B).

### Generation of neurons from parent and proband-specific iPSC lines

**Figure 1:**
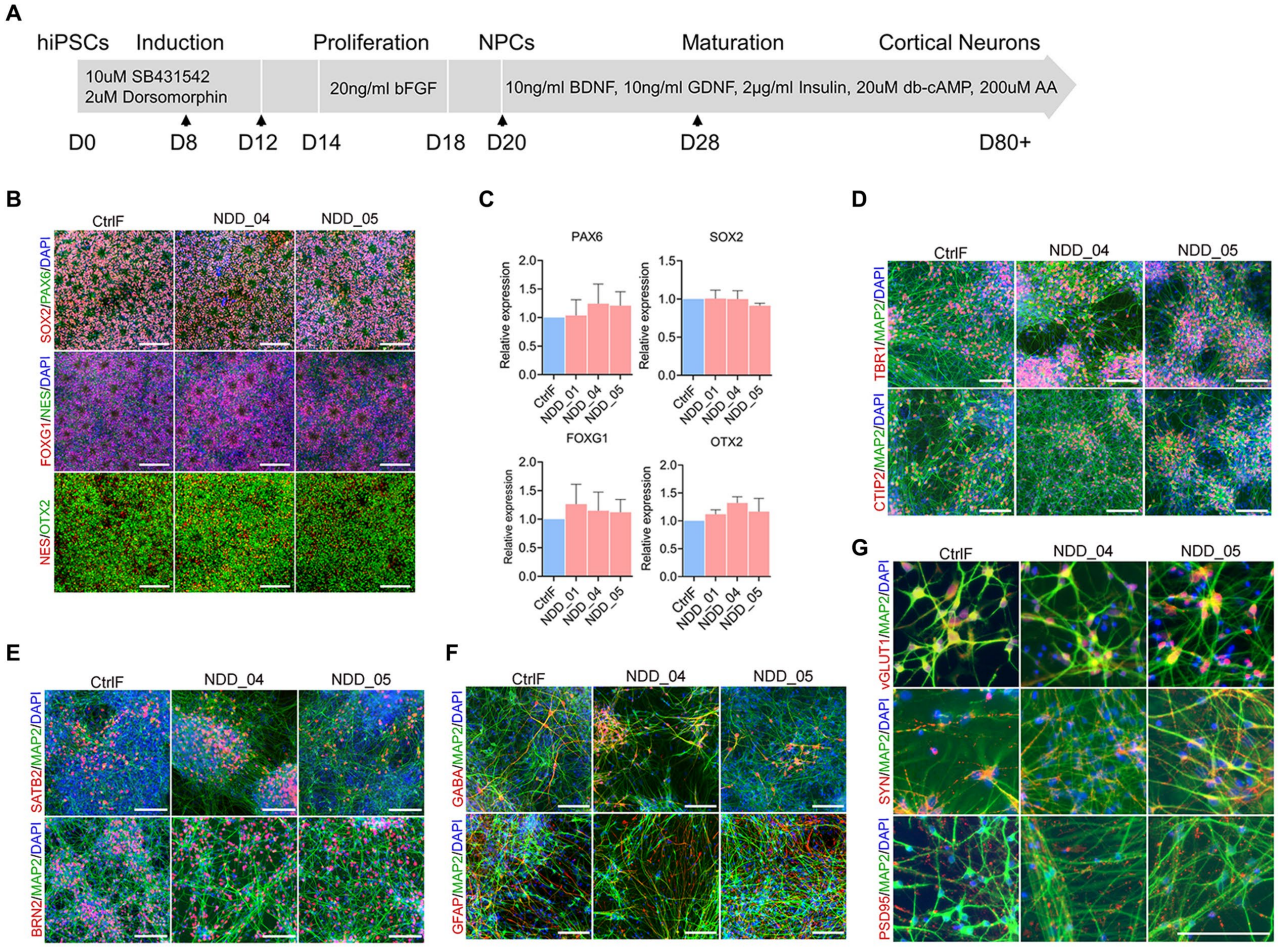
Differentiation of human induced pluripotent stem cell (iPSC) lines into cortical neurons. **(A)** Schematic representation showing the stepwise differentiation protocol, highlighting the small molecules and neurotrophic factors used during induction, proliferation, and maturation of cortical neurons. Arrow indicates the time of cell splitting. **(B)** Neural progenitor cell (NPC) immunostaining with neural progenitor markers PAX6, Nestin (Nes), SOX2, OTX2 and FOXG1 expression. **(C)** Quantification of progenitor markers expression using quantitative PCR (qPCR). There was no difference among multiple groups by using one-way analysis of variance (ANOVA) with Tukey test. Data were normalized to the housekeeping gene GAPDH. **(D–G)** Immunostaining of 8 week-old mature neurons with microtubule associated protein-2 (MAP2, a pan-neuronal marker), early born (deep layer) cortical neurons markers Tbr1 and CTIP2 **(D)**, later-born (upper layer) cortical neuron markers Brn2 (POU3F2) and Satb2 **(E)**, glial fibrillary acidic protein (GFAP) and GABA-positive GABAergic neurons markers **(F)**, vGlut1-expressing glutamatergic neurons, presynaptic markers synaptophysin (syn) and postsynaptic markers PSD95 **(G)**. Cell nuclei were stained with DAPI (blue). Scale, 100 μm. CtrlF: iPSC derived from father, NDD_01, NDD_04, and NDD_05: iPSC derived from probands.

Cortical neurons were produced by directed differentiation of the iPSC lines using a modified version of a published protocol (Shi et al., 2012) (Figure 1A). Briefly, neural progenitor cells (NPC) were generated from iPSC by dual inhibition of the SMAD signaling pathway (Chambers et al., 2009). After 12 days of neural induction the cells were expanded and proliferated in N2B27 neurobasal media supplemented with basic fibroblast growth factor (days 14–18). This approach produced self-organizing rosette structures with homogenous expression of neural progenitor markers PAX6, Nestin and SOX2 (Figure 1B). The cortical progenitor identity of NPC was verified by OTX2 and FOXG1 expression (Figure 1B; Supplementary Figure S1C). The expression of progenitor markers was quantified by qPCR. No differences were observed in control and mutant iPSC-derived NPC (Figure 1C), indicating efficient and equivalent neural conversion of wild type and mutant cells. NPC were differentiated further into mature cortical neurons by exposure to neurotrophic factors GDNF, BDNF, ascorbic acid, cAMP, and insulin for 8 weeks. Following differentiation, the iPSC-cortical neurons were immunostained to confirm their identity and maturation, and for the presence of glial cells. The early-born (deep layer) cortical neurons were identified by staining for TBR1 and CTIP2 transcription factors (Figure 1D; Supplementary Figure S1D), and the late-born (upper layer) neurons were identified by staining for transcription factor BRN2 (POU3F2) and SATB2 (Figure 1E; Supplementary Figure S1E), showing the efficient differentiation into cortical neurons with the anticipated characteristics of both upper and deep cortical layers. The cell type compositions of iPSC-derived neurons were characterized by MAP2, a pan-neuronal marker, staining for neurons and GFAP staining for astrocytes. Majority of neurons stained positive for MAP2, while 15%–20% of the cells were stained positive for GFAP (Figure 1F; Supplementary Figure S1F). The neuronal population also displayed both GABA-positive GABAergic neurons (Figure 1F) and vGlut1-expressing glutamatergic neurons (Figure 1G; Supplementary Figure S1G). Furthermore, neurons were positive for presynaptic markers synaptophysin (SYN) and postsynaptic marker PSD95, which colocalized with MAP2 (Figure 1G; Supplementary Figure S1G). In conclusion, iPSCs were efficiently differentiated into mature cortical neurons and no significant differences in differential potential were observed between the control and mutant iPSC-derived neurons.

### Loss of NAPB expression in proband-derived neurons

**Figure 2:**
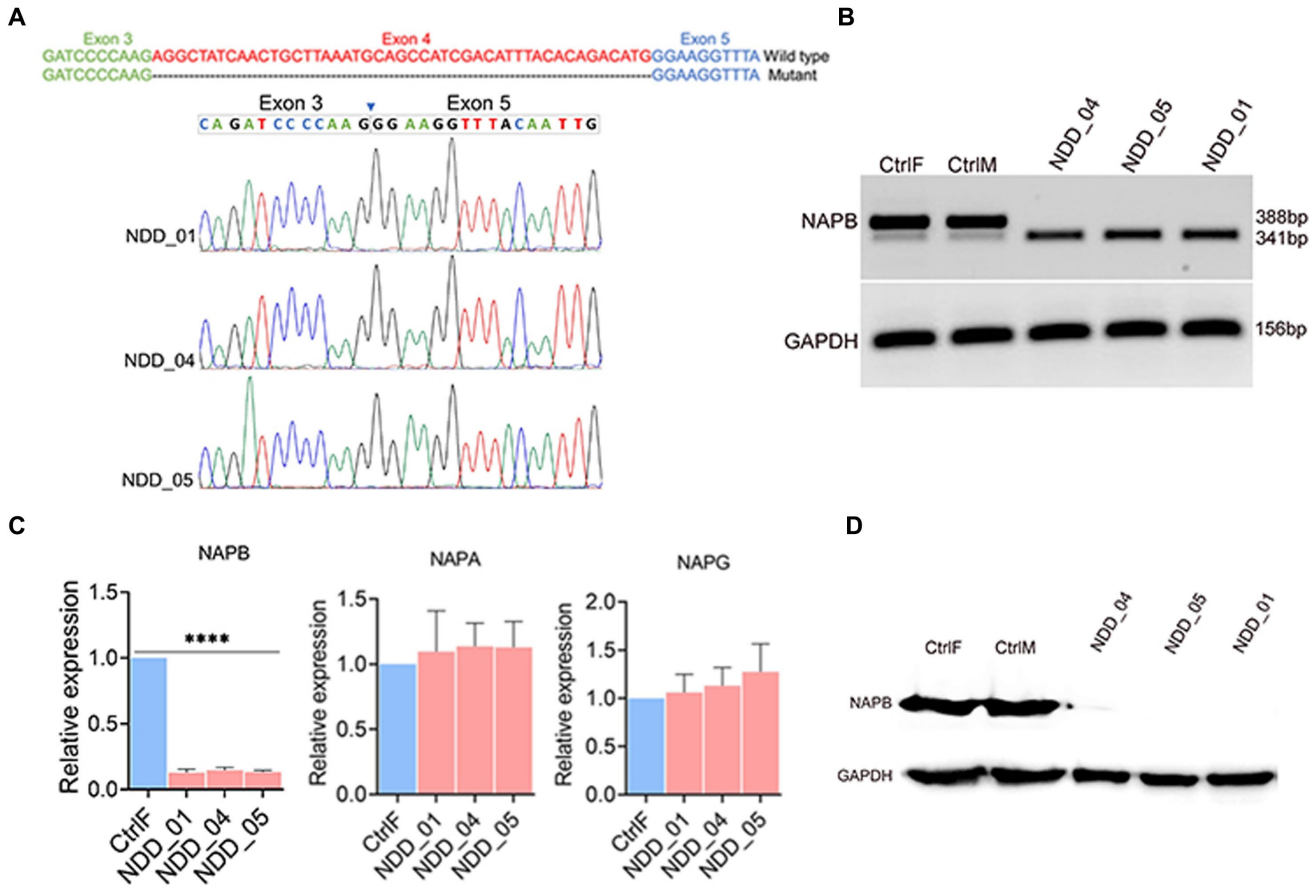
Validation of *NAPB* splice donor mutation (c.354+2T>G) in iPSC-derived cortical neurons. **(A)** Schematic of *NAPB* wild type and truncated mRNA sequences due to deletion of exon 4 (upper). Validation of exon 4 deletion in mutant cells by Sanger sequencing (bottom). **(B)** RT-PCR showing the expression of wild-type *NAPB* mRNA in control parents (heterozygous) and truncated transcript lacking exon 4 in probands (homozygous). **(C)** Expression quantification of *NAPB, NAPA* and *NAPG* mRNAs in control and mutant iPSC-derived cortical neurons using qPCR. Data are means ± SEM from 3 independent differentiation experiments. One-way ANOVA with Tukey test was used. ^****^p < 0.0001. **(D)** Western blot of NAPB protein (35 KDa) in control and proband iPSC-derived cortical neurons. GAPDH (37 KDa) was used as loading control. CtrlF and CtrlM: iPSC derived from father and mother, NDD_01, NDD_04, and NDD_05: iPSC derived from probands.

We assessed the expression of *NAPB* mRNA and protein in the iPSC-derived neurons from the parents and affected children. mRNA extracted from all 5 neuronal cultures was reverse-transcribed (RT), PCR amplified using primers from exons 3 and 5 that span the expected missing exon 4, and the amplicons were sequenced (Figure 2A). The RT-PCR results showed that neurons from the father and mother (CtrlF and CtrlM, respectively) expressed *NAPB* mRNA containing the 47 bp exon 4 (Figure 2B). The parents also expressed a low level of a shorter mRNA that lacked exon 4, thus confirming that the mutation we identified in a splice donor site results in the skipping of exon 4. These results indicate that NAPB is expressed from both alleles in the neurons derived from the heterozygous parents, mostly from the wild-type allele. In the probands, which are homozygous for the splice site loss variant, only the smaller amplicon was detected by RT-PCR and sequencing confirmed the absence of exon 4 (Figures 2A,B). Quantitative RT-PCR showed that the overall levels of NAPB mRNA was 10-fold lower in the neurons from the probands relative to the parents (Figure 2C; Supplementary Figure S2A). The NAPA and NAPG were also expressed in neurons and no difference in mRNA levels was detected in controls and probands (Figure 2C; Supplementary Figure S2A). Given the reduced levels of *NAPB* mRNA and the absence of a 47 bp exon in the probands, we then assessed NAPB protein expression in the derived neurons. Immunoblotting with a NAPB specific antibody detected a protein of the expected size (35 KDa) expressed in parent-derived neurons; this band was completely absent in the neurons from all three affected children (Figure 2D). Collectively, these results clearly establish that homozygous recessively inherited *NAPB* genetic variant results in a loss-of-function of NAPB in the neurons derived from the monozygotic triplets.

### Electrophysiological characterization of proband-derived neurons

**Figure 3:**
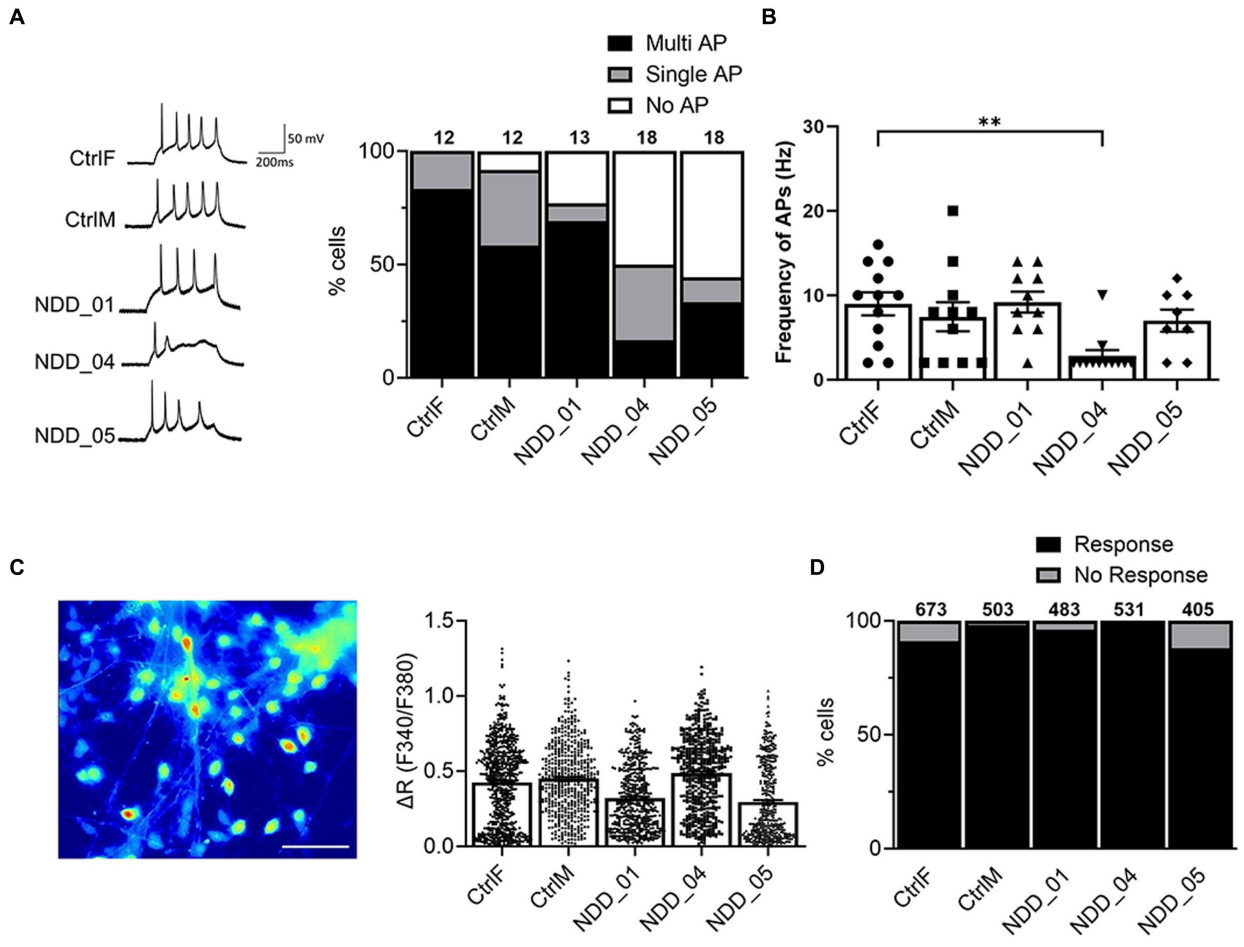
Electrophysiological characterization of iPSC-derived cortical neurons. **(A)** Whole-cell patch-clamp recording to monitor action potential (AP) generated by injection of a series of current steps from 20 to 60 pA for 500 ms in a current clamp mode; No AP, single AP, or multiple AP. **(B)** AP frequency (Hz), the number of spikes per second from 3 independent biological replicates: *n* = 12 (CtrlF), 12 (CtrlM), 13 (NDD_01), 18 (NDD_04), 18 (NDD_05). Data are means ± SEM and ordinary one-way ANOVA with Tukey’s multiple comparisons test was used. **(C)** Image of Fura-2-loaded iPSC-derived cortical neurons after 8 weeks of differentiation. Net changes of calcium increase by 50 mM KCl. Data are means ± SEM from 3 independent differentiation experiments. **(D)** Percentage of neurons that evoke calcium influx in response to 50 mM KCl. The number of cells tested are shown from 3 independent differentiation experiments: *n* = 673 (CtrlF), 503 (CtrlM), 483 (NDD_01), 531 (NDD_04), 405 (NDD_05). Evoked action potentials were recorded by a series of current steps from 20 to 60 pA for 500 ms in a current-clamp mode. CtrlF and CtrlM: iPSC derived from father and mother, NDD_01, NDD_04, and NDD_05: iPSC derived from probands. Scale, 20 μm.

We examined electrophysiological activities of the iPSC-derived neurons. Action potential (AP) generation is a functional hallmark of mature of neurons. We monitored AP in the current-clamp mode and distributions of AP generation were analyzed (no AP, single AP, or multiple/repetitive AP) in iPSC-derived cortical neurons in three independent differentiation experiments. Neurons from the parents and probands generated multiple and repetitive action potentials (Figure 3A). For two of the probands there was a trend towards fewer neurons with multiple AP which had somewhat reduced firing frequency (Figure 3A), however, there was no overall statistically significant difference between the wild-type and mutant neurons (Figure 3B).

We also examined functional activity of iPSC-derived neurons by single-cell calcium imaging using a Fura-2 ratiometric calcium indicator approach. Neurons express voltage-gated calcium channels (VGCC) that mediate calcium influx to trigger vesicle fusion and neurotransmitter release. We analyzed calcium influx through VGCC in iPSC-derived cortical neurons (Figure 3C). Most cortical neurons (>90%) evoked calcium influx upon 50 mM KCl stimulation, however no significant differences were observed between parent-derived and proband-derived neurons (Figure 3D). Collectively, the electrophysiological characterization of the neurons showed that our differentiation methods produce functional neurons, however we were unable to show significant differences in AP generation or calcium influx that correlated with the loss of NAPB function.

### Transcriptomic profiling of proband-derived neurons

**Figure 4:**
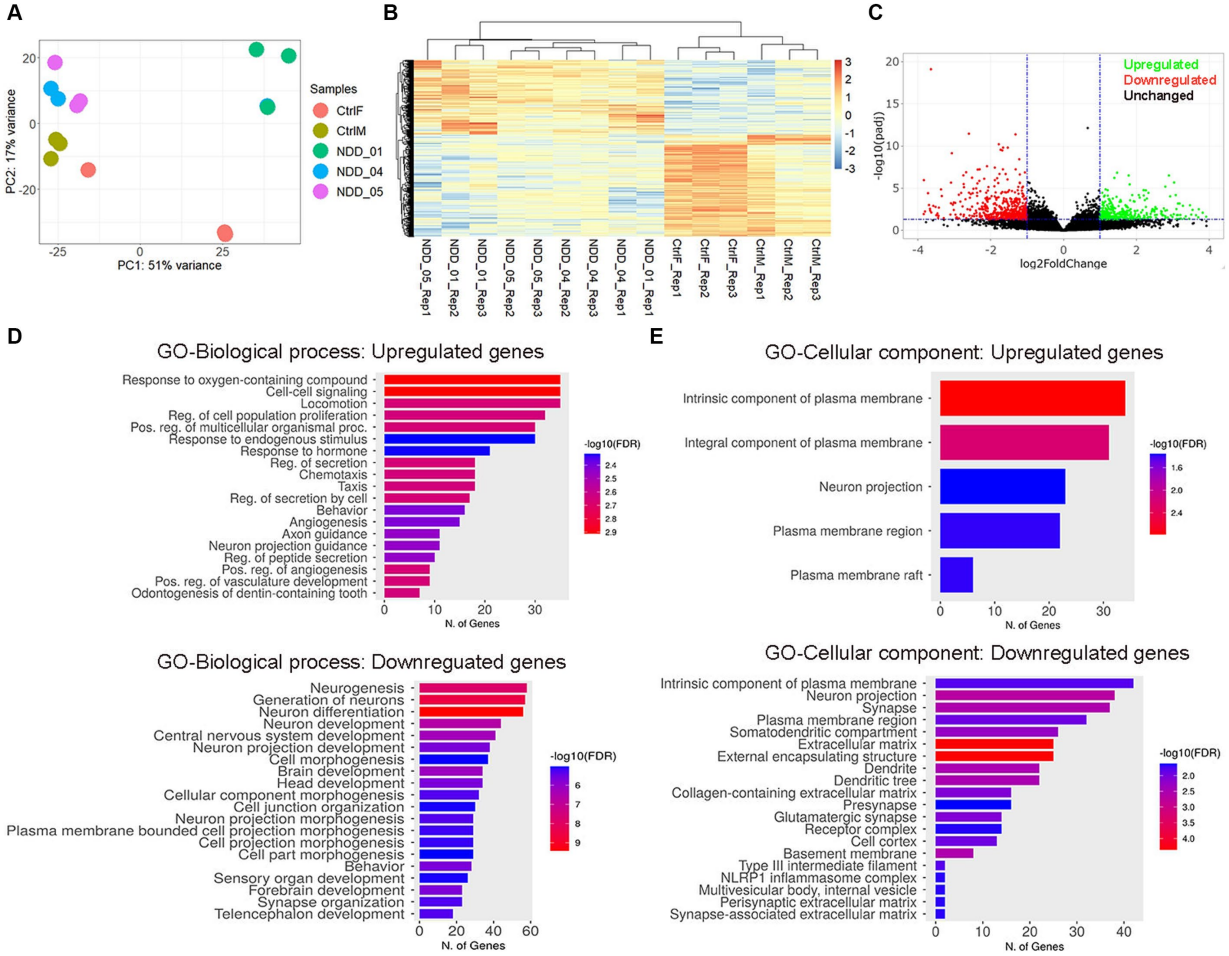
Transcriptome profiling of iPSC-derived cortical neurons. **(A)** Principal component analysis. Each point represents an RNA-Seq sample and samples with similar gene expression profiles are clustered together. Sample groups are indicated by using different colors as indicated in the legend provided. **(B)** Heatmap of hierarchical clustering analysis of all differentially expressed genes (DEGs) in mutant iPSC-derived cortical neurons, compared to control. Three independent biological replicates from each sample were analyzed (*p* < 0.05 and 2-fold change). Expression data were standardized as row *Z*-scores for each mRNA. **(C)** Volcano plot showing the log2 fold change and the adjusted *p*-value for all the detected transcripts; upregulated (green), downregulated (red), unchanged (black). **(D,E)** Gene Ontology (GO) enrichment analysis for biological processes and cellular components of upregulated and downregulated genes in iPSC-derived cortical neurons. The GO cut-off criteria included q (adjusted *p*-value) <0.05. CtrlF and CtrlM: iPSC derived from father and mother, NDD_01, NDD_04, and NDD_05: iPSC derived from probands.

**Figure 5:**
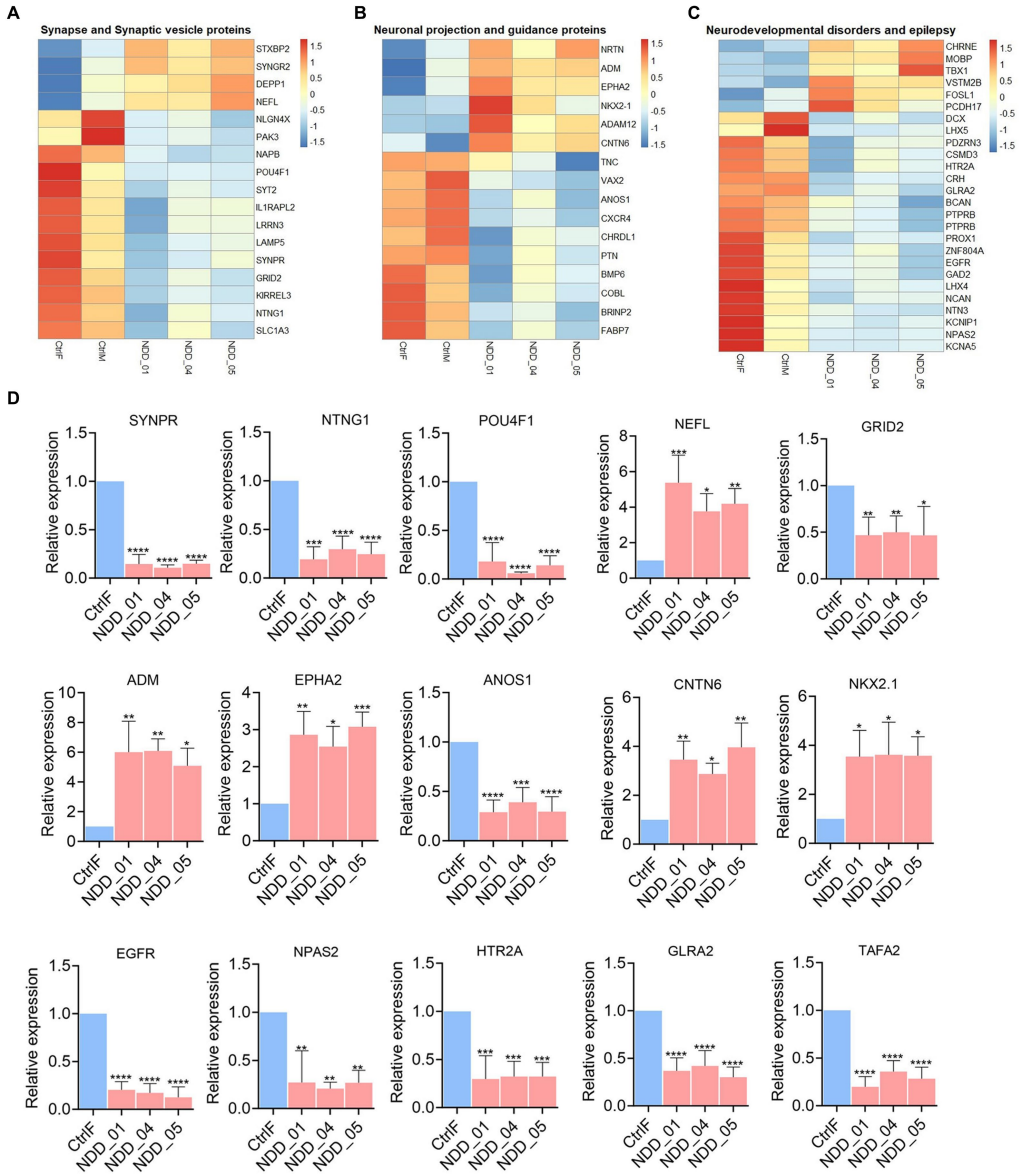
Sub-heatmaps and qPCR of differential expressed genes (DEGs) in mutant iPSC-derived cortical neurons. **(A–C)** Sub-heatmaps showing DEGs associated with synapse and synaptic vesicles **(A)**, neuronal projection and guidance **(B)**, neurodevelopmental disorders and epilepsy **(C)**. Each row represents mRNA transcript, and each column corresponds to a sample. Normalized row *Z*-score of mRNA abundance is depicted by a color scale with red indicating positive expression and blue indicating negative expression. **(D)** qPCR validation of the genes dysregulated in RNA-seq results. Graphs show mean with ±SEM of 3–4 independent biological replicates and the data were analyzed using one-way ANOVA with Tukey test. CtrlF and CtrlM: iPSC derived from father and mother, NDD_01, NDD_04, and NDD_05: iPSC derived from probands. ^*^p < 0.05, ^**^p < 0.01, ^***^p < 0.001, and ^****^p < 0.0001.

To understand the molecular effect of the *NAPB* mutation in cortical neurons, we performed transcriptome profiling to compare global gene expression in neurons derived from the parents and the affected children in three independent biological replicates. RNA was collected from 8 weeks-old neurons and analyzed by RNA sequencing (RNA-seq). Deep sequencing (approximately 20 million reads per sample) provided a comprehensive view of transcriptional differences between the probands and the parents (Figure 4). Principle component analysis demonstrated good reproducibility of the experimental replicates and variance in PC2 for the parent versus the probands (Figure 4A). Comparison of controls and probands identified 686 differentially expressed genes (DEGs) (*p*-value <0.05) of which 258 were upregulated [fold change (FC) >2] and 428 were downregulated [fold change (FC) < 2] (Figures 4B,C). Gene ontology (GO) enrichment analysis was performed (cut-off criteria of adjusted *p*-value <0.05) to identify biological processes/cellular component associated with DEGs. The key biological process terms for upregulated genes showed their role in cell–cell signaling, locomotion, regulation of secretion, behavior, axon guidance, neurons projection guidance and others. The downregulated genes were involved in neurogenesis, neuronal differentiation, behavior, forebrain development and synapse organization etc. (Figure 4D). The cellular component for the upregulated genes showed enrichment for GO terms including intrinsic and integral component of plasma membrane and neuron projection, while the downregulated genes showed enrichment for GO terms including intrinsic component of plasma membrane, neuron projection, synapse, extracellular matrix, and presynapse etc. (Figure 4E). These results demonstrate the impact of altered NAPB protein in disrupting critical processes involved in neurodevelopment, synapse organization, and neuronal functions. To confirm the RNA-seq data, we validated altered gene expression by qPCR for selected genes annotated in GO and previously reported to have role in synapse and synaptic vesicles (Figure 5A), neuronal projection and guidance (Figure 5B), neurodevelopmental disorders and epilepsy (Figure 5C). The qPCR confirmed the significant upregulation of *NEFL*, *ADM*, *EPHA2*, *CNTN6*, and *NKX2.1*, while *SYNPR*, *NTNG1*, *POU4F1*, *GRID2*, *ANOS1*, *EGFR*, *NPAS2*, *HTR2A*, *GLRA2*, and *TAFA2* were significantly downregulated, which were consistent with the RNA-seq data (Figure 5D; Supplementary Figure S2B).

### Correction of NAPB mutation in iPSC lines

**Figure 6:**
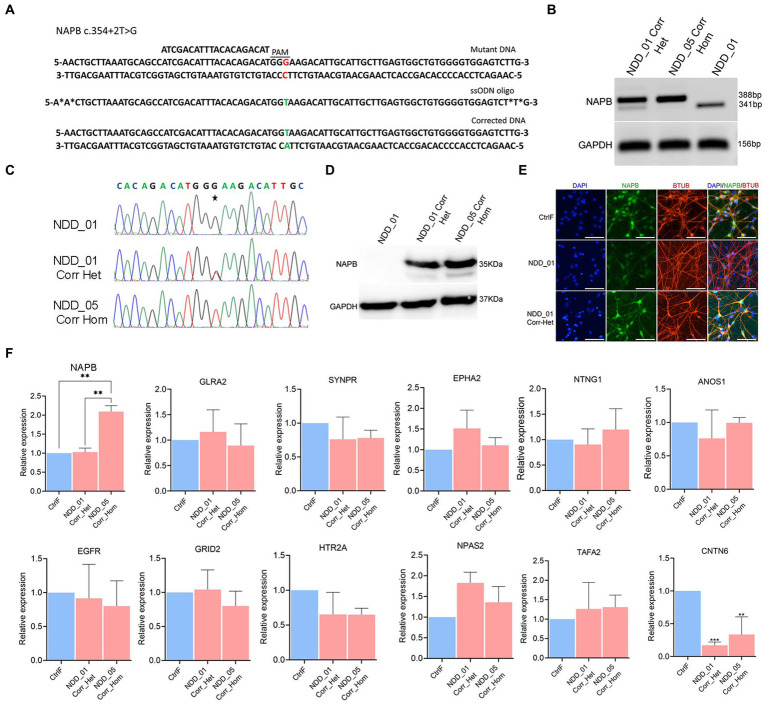
Correction of *NAPB* mutation (c.354+2T>G) using CRISPR/Cas9 gene editing. **(A)** Schematics of *NAPB* genomic region (top) having T to G nucleotide mutation (highlighted in red) with protospacer (ATCGACATTTACACAGACAT) sequence and protospacer associated motif (PAM, dashed line). Sequence of ssODN (middle, 82 nucleotides long) repair template with wild type T nucleotide (highlighted in green) carrying two phosphorothioate linkages at both 5′ and 3′ ends. Corrected DNA (bottom) following CRISPR/Cas9 editing with T nucleotide (highlighted in green). **(B)** RT-PCR of *NAPB* mRNA following mutation correction. GAPDH was used as loading control. The size of PCR product in mutant samples is 341 bp and 388 bp in corrected samples showing inclusion of 47 bp exon 4. **(C)** Sequence of genomic DNA following CRISPR/Cas9 editing in two clones of corrected probands. Examples of single cell derived clones having heterozygous and homozygous correction are shown. **(D)** NAPB western blot in mutant and corrected probands showing restoration of NAPB protein (35 KDa). GAPDH (37 KDa) was used as loading control. **(E)** Immunostaining of NAPB in control, mutant and corrected iPSC-derived cortical neurons. **(F)** qPCR validation of gene expression in corrected iPSC-derived neurons. Graphs show mean with ±SEM of 3–4 independent biological replicates and the data were analyzed using one-way ANOVA with Tukey test. CtrlF: iPSC derived from father, NDD_01: iPSC derived from proband, NDD_01 Corr_het: iPSC having heterozygous correction of NAPB mutation in NDD_01 proband, NDD_05 Corr_Hom: iPSC having homozygous correction of NAPB mutation in NDD_05 proband. ^*^p < 0.05, ^**^p < 0.01, ^***^p < 0.001, and ^****^p < 0.0001. Scale, 100 μm.

To correct the *NAPB* splice donor mutation in iPSC by CRISPR/Cas9-mediated gene editing, we used mutation specific sgRNA in combination with single stranded oligodeoxynucleotide (ssODN) as the homology directed repair template (Figure 6A). Following electroporation and growth of single cell-derived clones, the region of interest surrounding the mutation was sequenced. Analysis of the results confirmed successful correction of *NAPB* mutation in the two isogenic iPSC lines, generating both heterozygous and homozygous clones (Figures 6B,C). The CRISPR-corrected iPSC maintained the pluripotency characteristics of their parental iPSC lines. The proband and corrected iPSC were differentiated into mature cortical neurons. RT-PCR results confirmed restoration of NAPB mRNA transcript in corrected iPSC-derived neurons (Figures 6B,F). Immunostaining and immunoblotting confirmed the restored expression NAPB protein (Figures 6D,E).

We assessed the global gene expression profile in mutation-corrected iPSC-derived neurons by performing RNA-seq analysis. We first made a comparison between the corrected (both heterozygous and homozygous) and probands iPSC derived neurons (Supplementary Figure S3), which identified 1,018 DEGs (*p* < 0.05, FC >2), of which 529 were upregulated and 489 were downregulated in probands iPSC derived neurons (Supplementary Figure S3B). GO enrichment analysis (cut-off criteria of adjusted *p*-value <0.05) of downregulated genes show enrichment in key biological processes (cell–cell signaling, synaptic and trans-synaptic signaling, regulation of ion transport, behavior, modulation of chemical synaptic transmission etc.) and molecular function (signaling receptor activity, molecular transducer activity, transmembrane signaling receptor activity, ion channel activity, neurotransmitter receptor activity, voltage-gated cation channel activity etc.) (Supplementary Figure S3C).

Comparison of controls (CtrlF and CtrlM) versus heterozygous and homozygous corrected iPSC derived neurons identified 375 differentially expressed genes: 245 upregulated and 132 downregulated (*p* < 0.05, FC >2) (Supplementary Figures S4A,B). We then examined the impact of the NAPB correction on the genes that were differentially expressed between parents and proband derived cortical neurons. The results showed that the expression of 178 unregulated and 280 downregulated genes in mutant neurons were restored (69% and 66% respectively) in corrected iPSC-derived cortical neurons. The key biological process terms for upregulated genes in corrected cells showed their role in cell–cell signaling, response to endogenous stimulus, chemical synaptic transmission, synaptic signaling, behavior and others (Supplementary Figure S4C). Contrary to the mutated samples (Figure 4D), no biological processes were associated with downregulated genes in the corrected samples, indicating restoration of the processes involved in neurogenesis, neuronal differentiation, behavior, forebrain development and synapse organization. The restoration of gene expression in corrected neurons was validated by qPCR (Figure 6F; Supplementary Figure S4D). In conclusion, we successfully corrected *NAPB* mutation and showed the restoration of genes involved in synapse function, neurodevelopmental disorders and epilepsy. These iPSC lines will be useful to explore the molecular function of NAPB and how its dysfunction potentially contributes to the progression neurodevelopmental disorders.

## Discussion

We identified monozygotic triplets with epilepsy and autism who inherited two homozygous recessive genetic variants of interest from their consanguineous parents who were heterozygous for both genes: a missense coding variant in *VPS13B* and a splice site loss variant in *NAPB*. It is predicted that the *NAPB* variant is a splice site loss that would result in skipping of the 47 bp exon 4, thereby creating a frame shift that produces a truncated or completely untranslated NAPB protein. We hypothesized that loss of NAPB function was pathogenic and underlies the neurodevelopmental features displayed by the probands. To test this hypothesis, we have created a panel of iPSC lines from both parents and all three affected children, and we have generated isogenic iPSC lines from two of the probands with targeted correction of the *NABP* mutation. We have verified that the point mutation (c.354+2T>G) of *NAPB* does indeed cause exon skipping. We have shown that no NAPB protein is expressed in neurons derived from the probands’ iPSC lines but is expressed in the parents and mutation-corrected neurons (Figures 2D, 6D). Thus, we conclude that neurons of the affected triplets express no NAPB. Previous reports have identified NAPB mutations in children with early onset epilepsy that were predicted to be protein truncating (Conroy et al., 2016; Zhao et al., 2021; Mignon-Ravix et al., 2023). Here we are the first to provide experimental evidence for complete absence of NAPB in the neurons of probands carrying such genetic variants and provide experimental evidence for the role of NAPB protein in association with early onset epilepsy and autism spectrum disorders.

The *in vitro* generated cortical neurons harboring the *NAPB* mutation provides an excellent model system to evaluate the consequences of this loss of function. The system is amenable to comprehensive gene expression profiling. Hundreds of genes differentially expressed in mutant neurons relative to controls (parents and gene-corrected) were identified, many of which are associated with synapse and synaptic vesicles, neuronal projection and guidance, neurodevelopmental disorders and epilepsy (Figure 5). STXBP2 gene encodes Munc18-2 that belongs to the Sec/MUNC (SM) protein family, essential components of multiple intracellular membrane trafficking steps in eukaryotic cells (Sudhof and Rothman, 2009; Carr and Rizo, 2010). Synaptoporin is a component of the synaptic vesicle membrane (Jahn and Sudhof, 1999) and is expressed in telencephalic structures (Fykse et al., 1993). SYNGR2 plays an important role in cellular exocytosis, the storage and transport of glucose transporters (GLUT4) at the cytoplasmic membrane, and the formation and maturation of microvesicles in neuronal cells (Kioumourtzoglou et al., 2015). Synaptotagmin-2 (Syt2) is a synaptic vesicle membrane protein specific to inhibitory neurons and involved in fast, Ca2^+^ dependent neurotransmitter release (Ullrich et al., 1994; Pang et al., 2006a,b). Similarly, many genes involved in neurodevelopmental disorders, including *NPAS2*, *KCNIP1*, *LHX5*, *ZNF804A*, *HTR2A*, *GLRA2*, *CSMD3*, *PTPRB*, *EGFR* (Paylor et al., 2001; Xia et al., 2010; Abrahams et al., 2013; Paquette and Marsit, 2014; Galvez-Contreras et al., 2016; Ozburn et al., 2017) and epilepsy, including *DCX*, *CHRNE*, *GLRA2* and *KCNA5* etc. (Lapray et al., 2010; Becchetti, 2012; Kohling and Wolfart, 2016) were differentially expressed. These results show clear consequences in biological processes related to neurological functions, particularly in synapse and plasma membrane components (Figure 4), for which NAPB plays a known role.

The cortical neurons harboring the NAPB mutation also provide an excellent model system to perform basic electrophysiological measurements to compare wildtype and mutant neurons. Patch clamping studies showed multiple and repetitive action potentials emanating from neurons generated from the parents and probands (Figure 3A). There was a trend towards fewer neurons with multiple AP which had somewhat reduced firing frequency for the mutant neurons. However, there was no overall statistically significant difference between the wild-type and mutant neurons. The inherent variability of the iPSC clones and the *in vitro* differentiation process that takes 3 months are technical challenges to achieving statistically significant differences. It should also be noted that NAPB knockout mouse did not display any overt phenotypes unless NAPA was also knocked out (Burgalossi et al., 2010). Thus, more sensitive and focused functional assays are warranted to study the loss of NAPB function. The panel of well-characterized iPSC lines presented here provides a valuable resource for additional functional studies, particularly regarding the recycling of synaptic vesicles in cortical neurons and how its dysregulation contributes to clinical manifestations of epilepsy and autism.

## Data availability statement

The RNA-seq datasets generated and used in the present study are publicly available on the Zenodo repository: https://zenodo.org/records/10369577 and https://zenodo.org/records/10396611.

## Ethics statement

The studies involving humans were approved by the institutional review board in Hamad Bin Khalifa University (QBRI-IRB 2018-024) and all subjects were recruited with informed consent. The studies were conducted in accordance with the local legislation and institutional requirements. The participants provided their written informed consent to participate in this study.

## Author contributions

GoA: Conceptualization, Methodology, Visualization, Writing – original draft. KS: Data curation, Methodology, Writing – review & editing. WH: Project administration, Resources, Writing – review & editing. GhA: Methodology, Resources, Writing – review & editing. AA: Writing – review & editing. FA: Data curation, Formal analysis, Investigation, Writing – review & editing. YP: Conceptualization, Funding acquisition, Investigation, Methodology, Writing – original draft, Writing – review & editing. LS: Conceptualization, Funding acquisition, Project administration, Writing – original draft.

## Supplementary material

SUPPLEMENTARY FIGURE S1 **(A)** Sequence confirmation of NAPB (c.354+2T>G) and **(B)** VPS13B (c.8516G>A) mutation in heterozygous parents, wild type siblings and in triplets. **(C)** Neural progenitor cell (NPC) immunostaining with neural progenitor markers PAX6, Nestin (Nes), SOX2, OTX2 and FOXG1 expression. **(D-G)** Immunostaining of 8-week old mature neurons with microtubule associated protein-2 (MAP2, a pan-neuronal marker), early born deep layer cortical neurons markers Tbr1 and CTIP2 **(D)**, later-born upper layer cortical neurons markers Brn2 (POU3F2) and Satb2 **(E)**, glial fibrillary acidic protein (GFAP) and GABA-positive GABAergic neurons markers **(F)**, vGlut1-expressing glutamatergic neurons, presynaptic markers synaptophysin (syn) and postsynaptic markers PSD95 **(G)**. Cell nuclei were stained with DAPI (blue). CtrlF and CtrlM: iPSC derived from father and mother, NDD_01, NDD_04, and NDD_05: iPSC derived from probands. Scale, 100 µm.

SUPPLEMENTARY FIGURE S2 **(A)** Expression quantification of *NAPB, NAPA* and *NAPG* mRNAs in control mother (CtrlM) and mutant iPSC (NDD_01, NDD_04, and NDD_05) derived cortical neurons using qPCR. **(B)** qPCR validation of the genes dysregulated in RNA-seq results compared to control mother (CtrlM). Graphs show mean with ±SEM of 3-4 independent biological replicates. Data were analyzed using one-way ANOVA with Tukey test. **p* < 0.05, ***p* < 0.01, ****p* < 0.001, *****p* < 0.0001.

SUPPLEMENTARY FIGURE S3 Transcriptome profiling of iPSC-derived cortical neurons. **(A)** Principal component analysis. Each point represents an RNA-Seq sample and sample groups are indicated by using different colors as indicated in the legend provided. **(B)** Heatmap of hierarchical clustering analysis of all differentially expressed genes (DEGs) in mutant versus corrected iPSC derived cortical neurons. Three independent biological replicates from each sample were analyzed (*P* < 0.05 and 2-fold change). Expression data were standardized as row Z-scores for each mRNA. **(C)** Gene Ontology (GO) enrichment analysis for biological processes and molecular function of downregulated genes in mutant iPSC derived cortical neurons. NDD_01 Corr_het: iPSC having heterozygous correction of NAPB mutation in NDD_01 proband, NDD_05 Corr_Hom: iPSC having homozygous correction of NAPB mutation in NDD_05 proband.

SUPPLEMENTARY FIGURE S4 **(A)** Heatmap of hierarchical clustering analysis of all differentially expressed genes (DEGs) in corrected iPSC (NDD_01_Het and NDD_01_Hom) derived cortical neurons, compared to control (CtrlF and CtrlM). Three independent biological replicates from each sample were analyzed (*P* < 0.05 and 2-fold change). Expression data were standardized as row Z-scores for each mRNA. **(B)** Volcano plot showing the log2 fold change and the adjusted *P* value for all the detected transcripts; upregulated (green), downregulated (red), unchanged (black). **(C)** Gene Ontology (GO) enrichment analysis for biological processes of upregulated genes in corrected iPSC derived cortical neurons.GO enrichment analysis for cellular components of upregulated and downregulated genes in corrected iPSC-derived cortical neurons. **(D)** qPCR validation of genes expression in corrected iPSCs derived cortical neurons compared to control mother (CtrlM). Graphs show mean with ±SEM of 3–4 independent biological replicates. Data were analyzed using one-way ANOVA with Tukey test. NDD_01 Corr_het: iPSC having heterozygous correction of NAPB mutation in NDD_01 proband, NDD_05 Corr_Hom: iPSC having homozygous correction of NAPB mutation in NDD_05 proband. **p* < 0.05, ***p* < 0.01, ****p* < 0.001, *****p* < 0.0001.
