# Supplementary figures and images for: Characterization of a loss-of-function NSF attachment protein beta mutation in monozygotic triplets affected with epilepsy and autism using cortical neurons from proband-derived and CRISPR-corrected induced pluripotent stem cell lines
Source: Front Neurosci. 2024 Jan 8;17:1302470. doi: 10.3389/fnins.2023.1302470 (PMC10801733; doi:10.3389/fnins.2023.1302470)

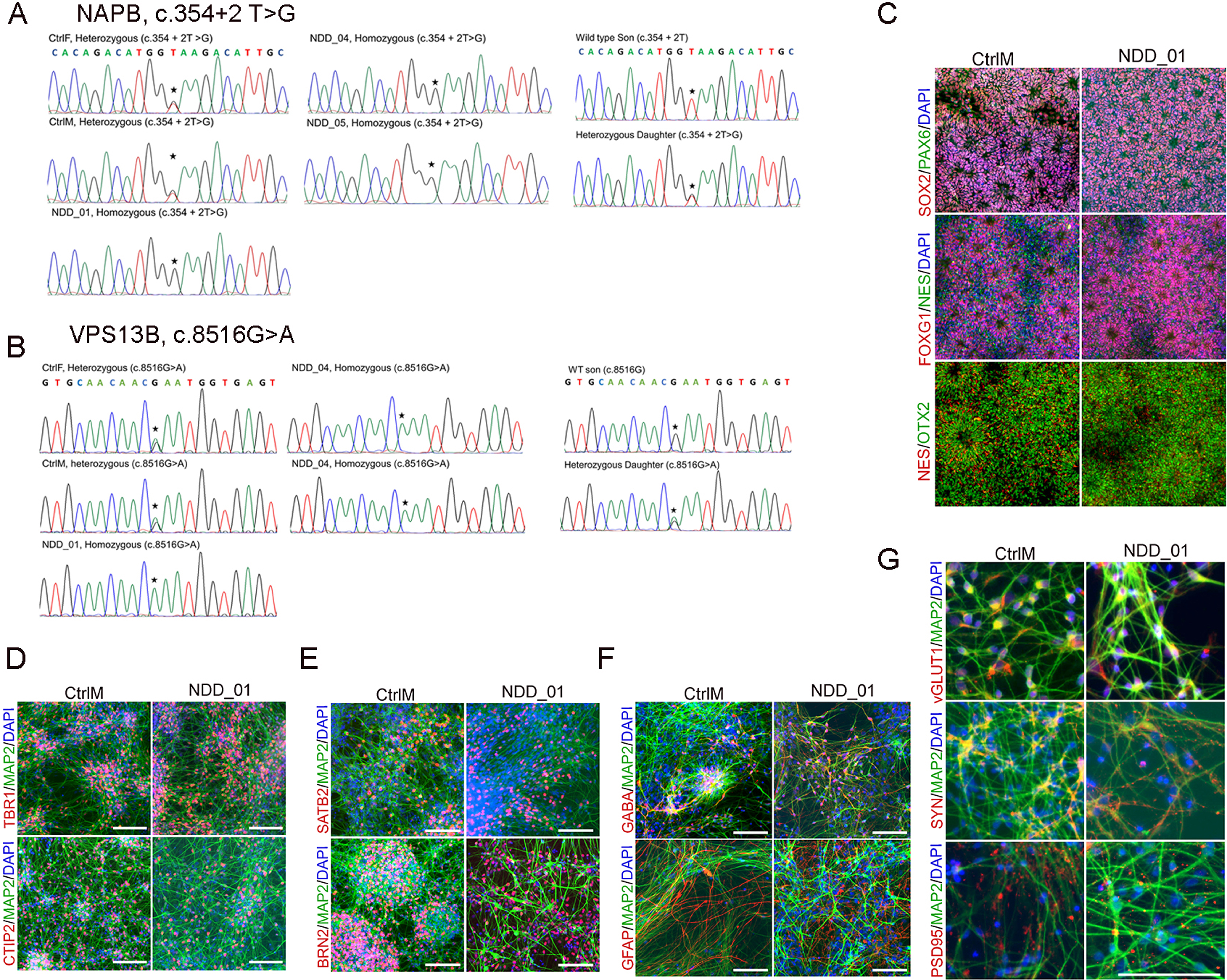

Supplement: SUPPLEMENTARY FIGURE S1 — (A) Sequence confirmation of NAPB (c.354+2T>G) and (B) VPS13B (c.8516G>A) mutation in heterozygous parents, wild type siblings and in triplets. (C) Neural progenitor cell (NPC) immunostaining with neural progenitor markers PAX6, Nestin (Nes), SOX2, OTX2 and FOXG1 expression. (D-G) Immunostaining of 8-week old mature neurons with microtubule associated protein-2 (MAP2, a pan-neuronal marker), early born deep layer cortical neurons markers Tbr1 and CTIP2 (D), later-born upper layer cortical neurons markers Brn2 (POU3F2) and Satb2 (E), glial fibrillary acidic protein (GFAP) and GABA-positive GABAergic neurons markers (F), vGlut1-expressing glutamatergic neurons, presynaptic markers synaptophysin (syn) and postsynaptic markers PSD95 (G). Cell nuclei were stained with DAPI (blue). CtrlF and CtrlM: iPSC derived from father and mother, NDD_01, NDD_04, and NDD_05: iPSC derived from probands. Scale, 100 µm. [file Image_1.JPEG]

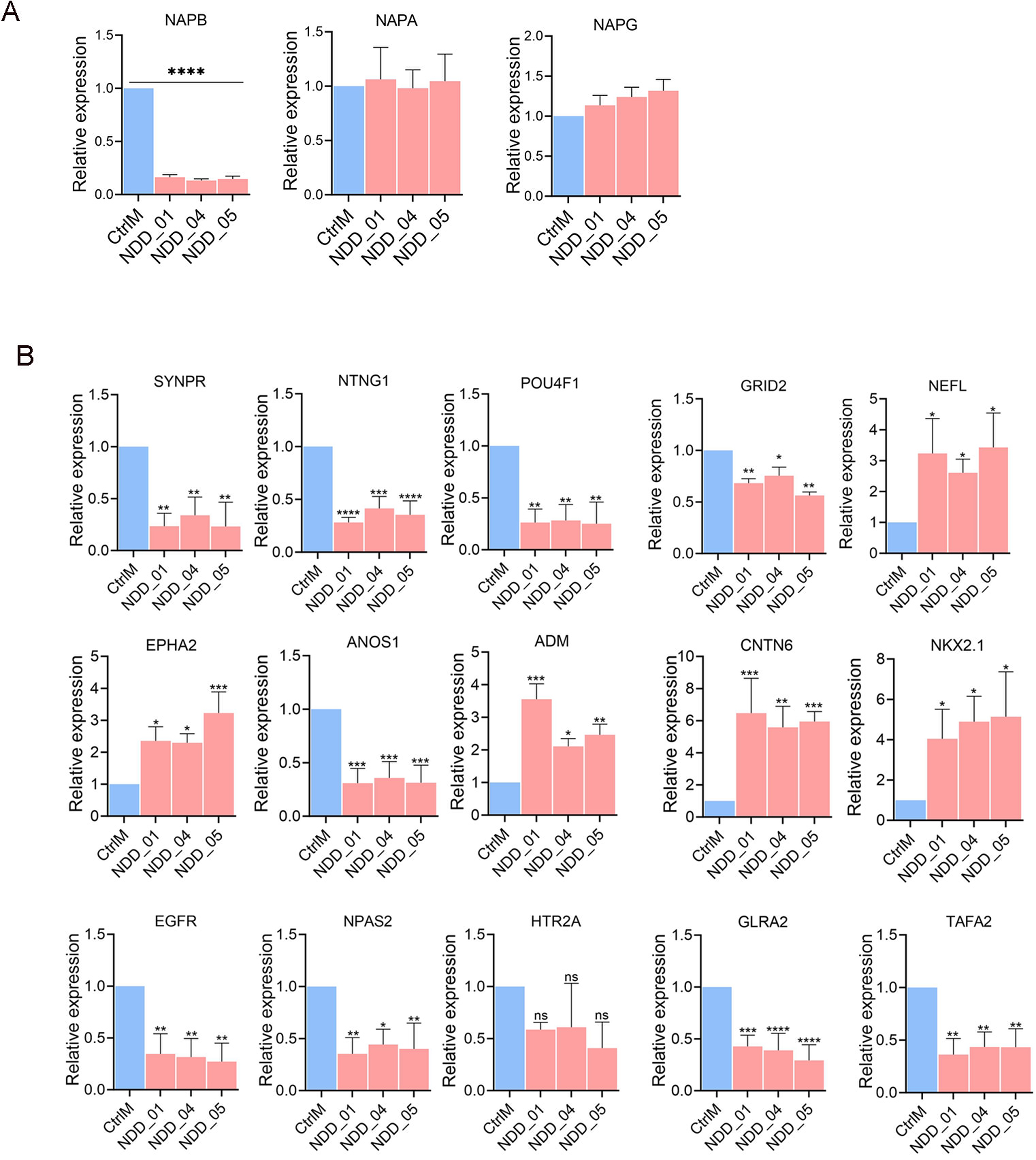

Supplement: SUPPLEMENTARY FIGURE S2 — (A) Expression quantification of NAPB, NAPA and NAPG mRNAs in control mother (CtrlM) and mutant iPSC (NDD_01, NDD_04, and NDD_05) derived cortical neurons using qPCR. (B) qPCR validation of the genes dysregulated in RNA-seq results compared to control mother (CtrlM). Graphs show mean with ±SEM of 3-4 independent biological replicates. Data were analyzed using one-way ANOVA with Tukey test. *p < 0.05, **p < 0.01, ***p < 0.001, ****p < 0.0001. [file Image_2.TIF]

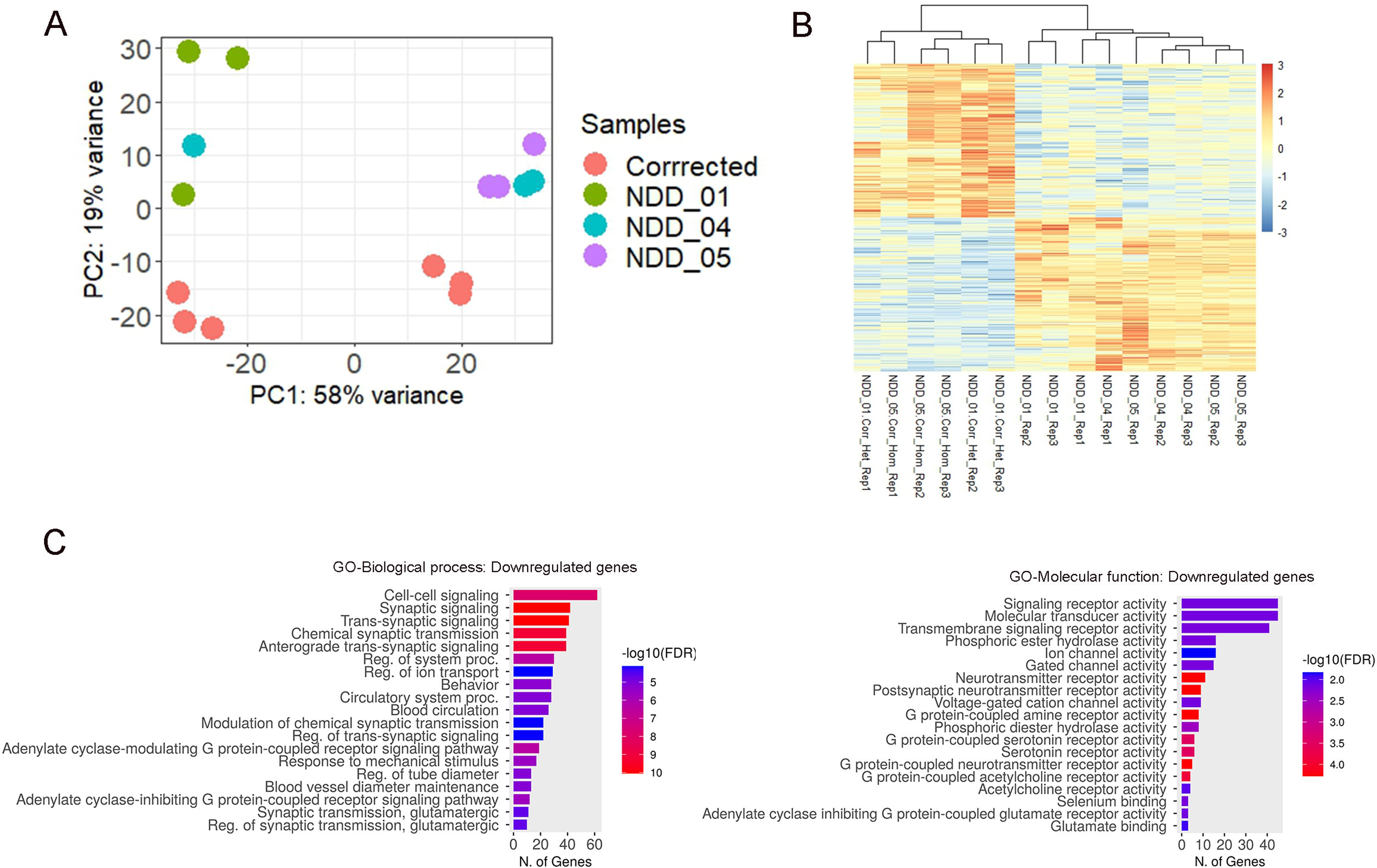

Supplement: SUPPLEMENTARY FIGURE S3 — Transcriptome profiling of iPSC-derived cortical neurons. (A) Principal component analysis. Each point represents an RNA-Seq sample and sample groups are indicated by using different colors as indicated in the legend provided. (B) Heatmap of hierarchical clustering analysis of all differentially expressed genes (DEGs) in mutant versus corrected iPSC derived cortical neurons. Three independent biological replicates from each sample were analyzed (P < 0.05 and 2-fold change). Expression data were standardized as row Z-scores for each mRNA. (C) Gene Ontology (GO) enrichment analysis for biological processes and molecular function of downregulated genes in mutant iPSC derived cortical neurons. NDD_01 Corr_het: iPSC having heterozygous correction of NAPB mutation in NDD_01 proband, NDD_05 Corr_Hom: iPSC having homozygous correction of NAPB mutation in NDD_05 proband. [file Image_3.TIF]

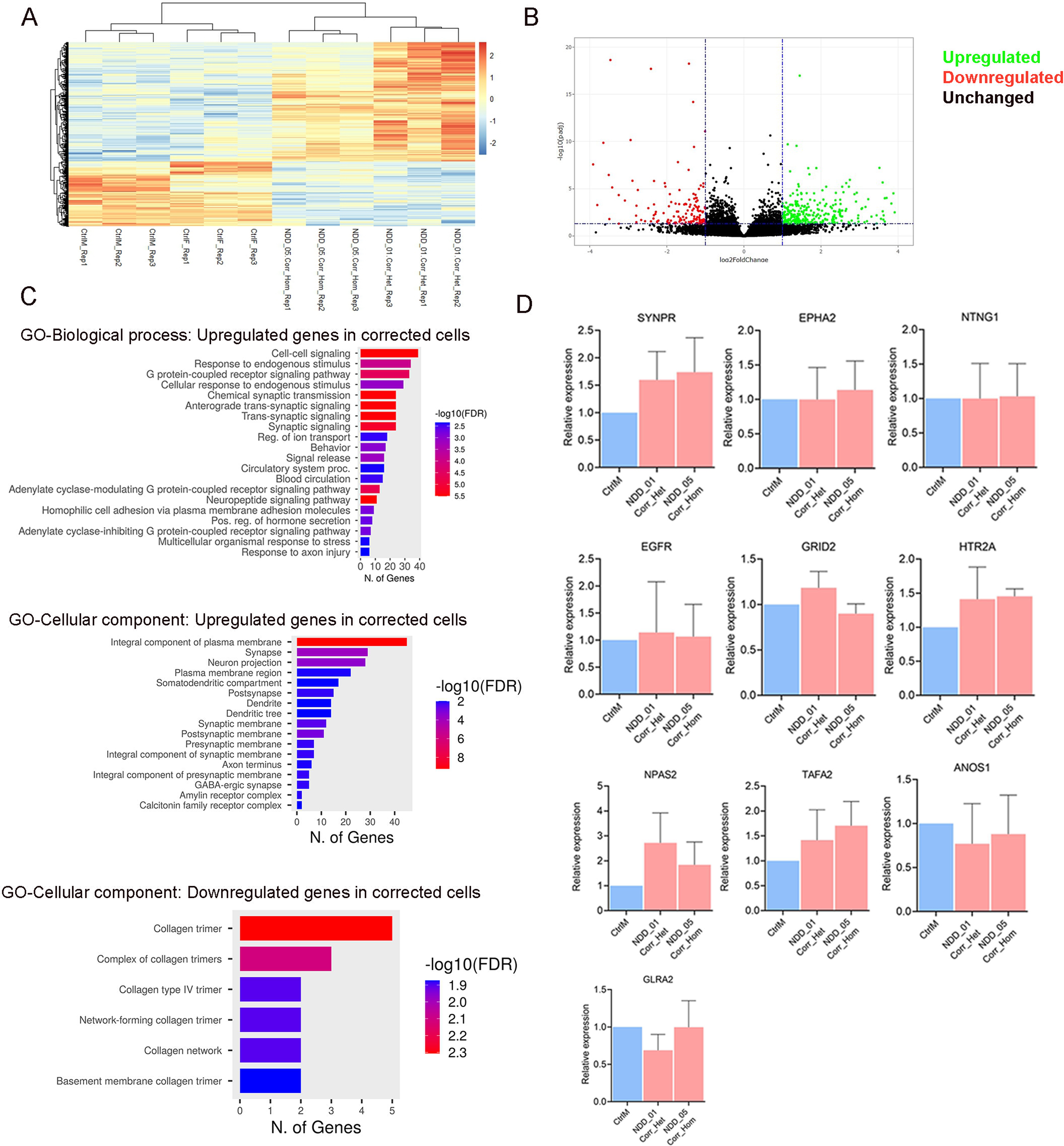

Supplement: SUPPLEMENTARY FIGURE S4 — (A) Heatmap of hierarchical clustering analysis of all differentially expressed genes (DEGs) in corrected iPSC (NDD_01_Het and NDD_01_Hom) derived cortical neurons, compared to control (CtrlF and CtrlM). Three independent biological replicates from each sample were analyzed (P < 0.05 and 2-fold change). Expression data were standardized as row Z-scores for each mRNA. (B) Volcano plot showing the log2 fold change and the adjusted P value for all the detected transcripts; upregulated (green), downregulated (red), unchanged (black). (C) Gene Ontology (GO) enrichment analysis for biological processes of upregulated genes in corrected iPSC derived cortical neurons.GO enrichment analysis for cellular components of upregulated and downregulated genes in corrected iPSC-derived cortical neurons. (D) qPCR validation of genes expression in corrected iPSCs derived cortical neurons compared to control mother (CtrlM). Graphs show mean with ±SEM of 3–4 independent biological replicates. Data were analyzed using one-way ANOVA with Tukey test. NDD_01 Corr_het: iPSC having heterozygous correction of NAPB mutation in NDD_01 proband, NDD_05 Corr_Hom: iPSC having homozygous correction of NAPB mutation in NDD_05 proband. *p < 0.05, **p < 0.01, ***p < 0.001, ****p < 0.0001. [file Image_4.TIF]
