# Supplementary material for: Characterization of a loss-of-function NSF attachment protein beta mutation in monozygotic triplets affected with epilepsy and autism using cortical neurons from proband-derived and CRISPR-corrected induced pluripotent stem cell lines
Source: Front Neurosci. 2024 Jan 8;17:1302470. doi: 10.3389/fnins.2023.1302470 (PMC10801733; doi:10.3389/fnins.2023.1302470)
Supplement: Supplementary file 5 [file Table_1.DOCX]

| **Gene** | **Forward primer 5ˈ-3ˈ** | **Reverse primer 5ˈ-3ˈ** |
| --- | --- | --- |
| PAX6 | CTACCTGAAGCAAGAATAC | CTACTGCTGATAGGAATATG |
| SOX2 | GGAGAGTAAGAAACAGCATGGA | GTGGATGGGATTGGTGTTCT |
| FOXG1 | CTTTACCCTGTGTTTATTTC | GTGCATTATAGTCACTTCTA |
| OTX2 | CTCACTCGCCACATCTACTTTG | GGTTTGGAGCAGTGGAACTTA |
| NAPA | GAGCTGTTCCCAGCTTTCT | GTCGTATTCCTTCACCGACTC |
| NAPB | CACAGACATGGGAAGGTTTAC | CTGAGCTGTTGGATTCTTCTC |
| NAPG | GTACGAGGACGTAGGTTTGATG | TCTGCAGCTACATAGTCATTTCT |
| SYNPR | ACTTCTGTGGTCTTTGGATTCT | AGTGCTTCTCCATTGGATCTG |
| NTNG1 | CGAGGAAGGTGCAAGTGTAA | AAGGTCGGCCCTGATAATTC |
| POU4F1 | CAACCAGAGACAGAAGCAGAA | AGGATAACGGACACTCCAAATC |
| NEFL | AAGAAGCTGCCAAGGAAGAG | CCCAGCACCTTCAACTTTCT |
| GRID2 | GGTGCTTCTGCTTGCTAATG | CCACTAACTCCACCCTTCTTG |
| ADM | TCACTCTCTTAGCAGGGTCT | CGACTCAGAGCCCACTTATT |
| EPHA2 | GGCAGGAGTTGGCTTCTTTA | GGGCTTCAGTTGTTCTGACT |
| ANOS1 | AGCGAGTTCAACTGACTGAC | GGGCAGATGGATCTTTGGAA |
| CNTN6 | ATCCCGAACTTCCAACAAGAA | CCCATTCTGGAGGAGCATAAA |
| NKX2.1 | TCGATGAGTCCAAAGCACAC | CTCCATGCCCACTTTCTTGTA |
| EGFR | CTTGCCGCAAAGTGTGTAAC | GAGATCGCCACTGATGGAG |
| NPAS2 | GTCTGGCAACACCACAATTC | GATTGGAGGTGCTCTGTGAT |
| HTR2A | TCAGTAGGTATATCCATGCCAAT | AAGAGCCGATCAGGACAAAG |
| GLRA2 | GTGCAAGTTGCTGAAGGATTG | GCAGGTAAACTTTCCAGTGTTG |
| TAFA2 | GCACTCCACAGATGCTGTAA | TCCACTATTGAAGCATCCACAC |
| NAPB genomic DNA | TTCTTGTGATGTGGTGCCATGTTC | CTCTCATGTCTTAGATGTCCACTCC |
| **NAPB Knock-in oligo and guide RNA** | **Sequence (5ˈ-3ˈ)** | |
| NAPB guide RNA | ATCGACATTTACACAGACAT | |
| NAPB Knock-in oligo | A*A*CTGCTTAAATGCAGCCATCGACATTTACACAGACATGGTAAGACATTGCATTGCTTGAGTGGCTGTGGGGTGGAGTCT*T*G | |

**Table S1**: Primer sequence of the genes for qPCR in the study
